# Abstract of Proceedings of the Buffalo Medical Association

**Published:** 1868-12

**Authors:** T. M. Johnson

**Affiliations:** Sec’y.


ART. III.—*Abstract of Proceedings of the Buffalo Medical Association.*

TUESDAY EVENING, NOV. 3d, 1868.

The meeting was called to order by the President. Members present—Drs. J. R. Lothrop, Rochester, White, Diehl, Wetmore, Ring, Miner and Johnson.

Dr. Diehl was elected Treasurer to fill the vacancy caused by the resignation of Dr. C. F. A. Nichell resigned.

DR. ROCHESTER presented a section of the stomach of a middle-aged man who consulted him a short time since, complaining of irritation and pain in the epigastric region. He recommended the man to go to the hospital, which he did, and died there a few days thereafter. The section exhibited, contained three polypoid growths. Their origin is within two or three inches of each other and near the pylorus. The largest was about an inch in its longest diameter. He presented the specimens because he believed it quite rare to see polypoid growths attached to the mucous membrane of the stomach.

DR. MINER remarked upon the probable rarity of such disease; had never seen it before, and was hardly aware that polypus had been observed in the stomach or bowels. Similar growths were very common, springing from mucous membranes near external orifices; the nasal polypus perhaps being most frequent. It had appeared to him probable that their appearance upon mucous surfaces near external parts, was due, in part at least, to irritations and other disturbances much more common in these exposed situations. The neck of the uterus furnished attachments to such growths much more frequently than the body of the organ, and it suffered from numerous sources of irritation not liable to affect the body. Nasal polypi, also, more frequently spring from the most exposed portions of the nasal cavity, but may, however, spring from any part of it. He had no fixed opinions concerning the causes of these growths, and his remarks were designed only as suggestive of possible causes.

Dr. Rochester said, it is difficult to determine the causes of these growths. Wherever they are found there is engorgement and irritation. In the case in question the man was and had been for a long time addicted to intemperance.

Dr. White said, I arise to bear testimony to the value of the specimen presented. It is peculiarly interesting to me, being the first of the kind I ever saw. Had seen many polypoid growths, but never before saw one arising from the mucous surface of the stomach or the intestinal canal. This brings up the whole subject of polypus. They are, I believe, of two kinds, viz: fibrous and mucus. Both of these varieties are frequently found in the uterus. The fibrous variety grow most frequently from the surface of the fundus and body, while the mucous variety grows most frequently from the mucous surface of the cervical portion. The fibrous polypus is usually larger but less irritating than the mucous. The mucous, although smaller and near the outlet, causes more irritation and hemorrhage and is more troublesome.

Dr. Lothrop thought that polypus of the larynx could not be accounted for upon any theory of mechanical irritation. Neither could it be assigned as the cause of polypi in many other localities. These growths appeared in situations in which the mucous membrane was liable to take on inflammation, and the inflammation was more likely the cause of the growths. The specimen presented by Dr. Rochester was interesting from the distinct character of the growths, one being the true mucous polypus and the other a fibroid concretion such as are sometimes found in submucous tissues of small size and projecting inwards. He supposed under favorable circumstances it would push outwards as well, and form a fibrous growth external to the stomach. He had never before seen a case in which these growths were found in the stomach, and thought it rare and of much interest.

Dr. Lothrop said that the large floodings which are common in cases of fibrous growths of the uterus, could not depend wholly upon the vascularity of the growths themselves, since they happen where from the situation of the growth it could not come from it. When they project into the cavity a portion of the bleeding was from them, but the same and even greater hæmorrhage would occur in cases when the growth was intramural.

Dr. White related the three following cases of removal of polypoid growths from the uterus:

Case I.—A clergyman from Ohio brought his wife to me, who had suffered several years with severe hæmorrhage and irritation, and was considerably reduced in health and strength. Gave chloroform, and on examination found a fibrous tumor, which I seized with hooked forceps, drew it down and cut it off with scissors. Applied glycerine and persulphate of iron when the hæmorrhage soon ceased. I have since learned that she has entirely recovered her health.

Case II.—A few days ago went to Genesee Station, and on examination of the case found a fibroid tumor protruding from the os uteri. Gave chloroform, drew down the tumor and cut it off as in the first case. The tumor was as large as an ordinary sized egg-plant. The pedicle was an inch and a half or two inches in diameter.

Case III.—Saw a woman a few days since who had consulted several physicians who had said that she had inverted uterus. On examination I found a fibroid tumor, which I removed as in the other cases mentioned, and the patient is rapidly recovering.

Formerly I applied a ligature in all cases and employed Gooche's instrument for that purpose. Afterwards used an instrument of my own invention for that purpose, but now do not use a ligature at all, but cut off the pedicle with long, curved scissors, and have no fear of dangerous hæmorrhage.

Dr. Miner related an instance of recent removal of a fibrous tumor springing from the uterine neck which had been of months standing, and the case treated and regarded as malignant disease. The growth had attained considerable size, and to careless observation might be taken for inverted uterus; it much more resembled it than any form of malignant disease. It had its origin from the lower part of the cervical canal, and had so attached itself to the neck as to leave the os upon the side of the pedicle where it was found after careful searching. The tumor would weigh about one pound and a half. Its union to the uterine neck was so complete that it would have been impossible to determine where the uterine neck ended and pedicle commenced, but for the os being found in the side. Hæmorrhage had been slight but constant for many weeks, and the patient looked completely blanched and bloodless. Upon making traction upon the growth the whole tumor was easily exposed to view and the uterine neck as well. The outer surface of the tumor was very vascular, and one or two small vessels gave off streams of blood with considerable force. Considering the value of every remaining drop of blood, a double ligature was passed and tied, after which the neck of the tumor was divided with the scissors. The interior of the tumor and neck where divided, contained few vessels, was fibrous in character, and doubtless, as Prof. White had suggested, might have been safely divided without the previous application of the ligature. The ligature could hardly be expected to do great harm and in such case might be useful. The physician in attendance had informed him that the patient was doing well.

Dr. Rochester said, in regard to prevailing diseases, that he had seen several cases of fever commencing with unusual symptoms, great irritability of the stomach, continuing several days, then followed by very severe symptoms.

Adjourned. T. M. Johnson, Sec'y.

